
JOURNAL INFORMATION
==============================
NLM Title Abbreviation: ScientificWorldJournal
Journal ID: ScientificWorldJournal
NLM Journal ID: 101131163
Journal ID: TSWJ

The Scientific World Journal

ISSN: 2356-6140
EISSN: 1537-744X
Publisher: Wiley

ARTICLE INFORMATION
==============================
PMCID: PMC6083966
PMID: 30147595
DOI: 10.1100/tsw.2001.217
Article ID: 356805
Article version: 1
Subjects: Short Report

Telomerase and Cell Immortality: Applications in Research and Medicine

Harley Calvin B.
Chief Scientific Officer, Geron Corporation, Menlo Park, CA, 94025, USA
Publication date: 2001
Electronic publication date: 2001 Nov 2
Volume: 1
First page: 115
Last page: 115
Copyright: Copyright © 2001 Calvin B. Harley.
Copyright year: 2001
License: This is an open access article distributed under the Creative Commons Attribution License, which permits unrestricted use, distribution, and reproduction in any medium, provided the original work is properly cited.
License URL: https://creativecommons.org/licenses/by/3.0/

==============================
Miami *Nature Biotechnology* Short Reports
*TheScientificWorld* (2001) 1 (S3), 115SR
ISSN 1532-2246; DOI 10.1100/tsw.2001.217

### TELOMERASE AND CELL IMMORTALITY: APPLICATIONS IN RESEARCH AND MEDICINE

Calvin B. Harley

Chief Scientific Officer, Geron Corporation, Menlo Park, CA, 94025

Telomerase is a reverse transcriptase that synthesizes telomeric DNA, the "mitotic clock" of replicative senescence in normal human somatic cells (Fig. 1,2). Telomerase is repressed in most normal human somatic cells but is active in immortal cells within the reproductive lineage and within tumors (Fig. 3). Inhibition of telomerase for applications in oncology has been well documented elsewhere and will not be reviewed here. However, applications of telomerase activation have not been as fully explored and will be the focus of this presentation.

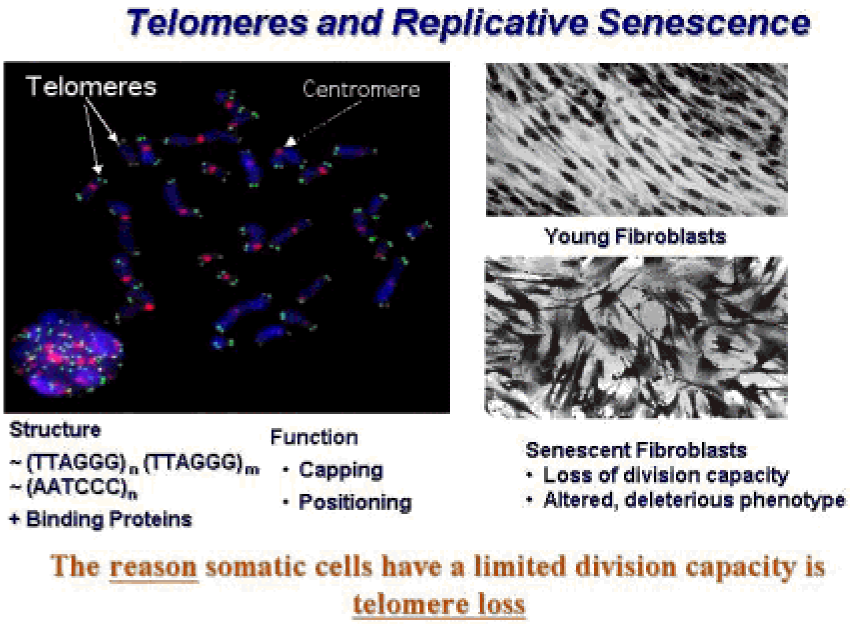

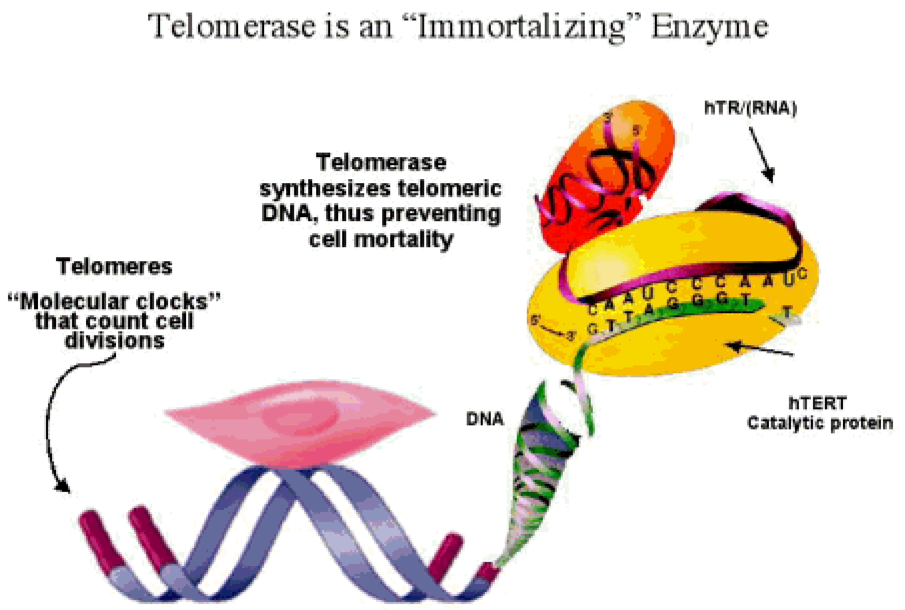

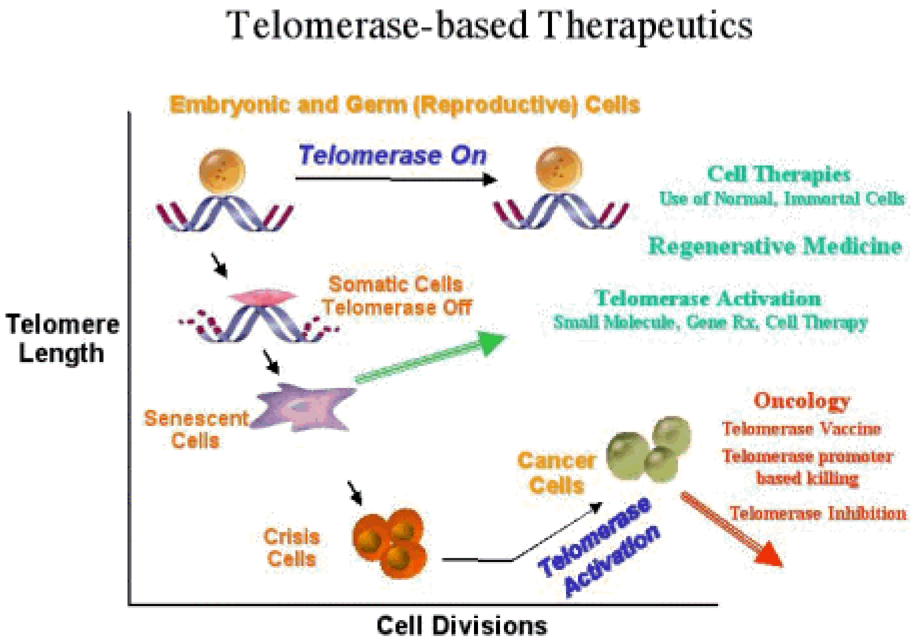

Forced expression of the limiting telomerase reverse transcriptase catalytic protein (hTERT) in several telomerase-negative normal human cell types is capable of indefinitely extending the lifespan of the cells (Fig. 3). It does so without loss of genomic stability, growth response signaling, or normal differentiation capacity. Moreover, the youthful phenotype, as measured by gene expression analysis and by function, is predominantly maintained in telomerase transduced cells. Variations in phenotype from the normal pattern in some telomerase transduced cell populations are known, but these have not been shown to be associated with loss of function or neoplastic transformation. The biological significance of such changes, which may be related to the relatively high levels of hTERT in transduced cells, remains to be determined.

Telomerase was reported to be insufficient for immortalization of certain epithelial cell types, including keratinocytes, as deregulation of cell cycle control mechanisms, particularly p16ARF, was also required. We and others have found that under appropriate tissue culture conditions telomerase alone immortalizes keratinocytes while retaining normal cell cycle control functions, including p16, response to positive and negative growth conditions, and differentiation capacity. Telomerase-transduced keratinocyte populations from multiple neonatal and adult skin donors have lifespans that now exceed the control populations by 5-10 fold.

There are multiple applications for telomerase-immortalized or extended-lifespan normal human cell populations (Fig. 4). These include basic and applied research applications such as investigations of other aspects of the normal cellular aging process, disease modeling, and cells for drug discovery assays including efficacy and safety testing. In addition, telomerase immortalized normal human cells have a lifespan which permits multiple rounds of genetic engineering. Thus, a variety of transgenic cell populations should also prove useful in production of biological materials such as vaccines and recombinant therapeutic proteins. Use of a immortalized human cells in these applications has advantages over use of animal cells or tumor cell lines owing to maintenance of normal human signaling pathways and post-translational modifications of targeted proteins, as well as safety factors. Telomerase extended lifespan cells produced ex vivo may also find application in cell or tissue transplantation therapies. Examples include the use of immune cell subpopulations for immunodeficiencies and cancer, skin cells for ulcers or burns, endothelial cells for vascular diseases, engineered hematopoietic or muscle cells for gene therapies, and retinal pigmented epithelial cells for macular degeneration.

Cell and Gene Therapy Disease Applications

✓ – **Fibroblasts/Keratinocytes/Endothelial Cells:** Skin Conditions

✓ – **Endothelial Cells:** Angiogenesis

✓ – **Smooth Muscle Cells:** Vascular diseases

✓ – **RPE Cells:** Macular Degeneration

✓ – **T Cells:** Infectious Diseases, AIDS, and Cancer

? – **Hepatocytes:** Chronic Liver Disease

? – **CD34 Cells:** Bone Marrow Transplantation and Gene Therapy

– **Chondrocytes:** Osteoarthritis, Joint Diseases

– **Follicular mesenchymal stem cells:** Hair restoration

Direct telomerase activation in vivo should also have significant medical application in degenerative diseases in which focal replicative senescence due to chronic stress or aging plays a role. Transient telomerase activation by gene therapy in vivo for the treatment of degenerative conditions such as liver disease, ulcers, or immunodeficiencies may be the first practical approach. In the longer term, pharmacologic activation of endogenous telomerase should be possible with small molecule drugs. It is already known that certain normal human cell lineages in tissues with high proliferative capacity are competent for transient activation of telomerase following specific environmental signals.

Although there are still research questions to be addressed, the exciting industrial and therapeutic potential of telomerase modulation in normal human cells clearly warrants diligent pursuit today.
